# Marker-trait association analysis for root and shoot traits at the seedling stage of wild barley (*Hordeum vulgare* subsp. *spontaneum*) under water stress and normal conditions

**DOI:** 10.1093/aobpla/plaf022

**Published:** 2025-07-16

**Authors:** Hooman Shirvani, Ali Ashraf Mehrabi, Mohsen Farshadfar, Hooshmand Safari, Ali Arminian, Foad Fatehi

**Affiliations:** Department of Agronomy and Plant Breeding, Faculty of Agriculture, Ilam University, Pajoohesh Boulevard, Ilam 69318-51147, Iran; Department of Agronomy and Plant Breeding, Faculty of Agricultural Sciences, Shahed University, Khalij Fars Expressway, Tehran 3319118651, Iran; Forests and Rangelands Research Department, Kermanshah Agricultural and Natural Resources Research and Education Center, Agricultural Research, Education and Extension Organization (AREEO), Jam Jam Blvd., Pasdaran Square, Kermanshah 67158-48333, Iran; Forests and Rangelands Research Department, Kermanshah Agricultural and Natural Resources Research and Education Center, Agricultural Research, Education and Extension Organization (AREEO), Jam Jam Blvd., Pasdaran Square, Kermanshah 67158-48333, Iran; Department of Agronomy and Plant Breeding, Faculty of Agriculture, Ilam University, Pajoohesh Boulevard, Ilam 69318-51147, Iran; Department of Agriculture, Payame Noor University, Nakhl Street, Lashkarak Highway, Tehran 19395-4697, Iran

**Keywords:** crop wild relatives, germplasm, root, marker–trait association

## Abstract

Wild barley (*Hordeum vulgare* subsp*. spontaneum*), the progenitor of cultivated barley, is an invaluable genetic resource for enhancing crop resilience, particularly in drought-prone regions. Its natural adaptation to water-limited environments makes it an ideal candidate for studying mechanisms of drought tolerance. This study aims to investigate the genetic basis of drought tolerance by examining the correlation between molecular markers and root traits across a diverse collection of wild barley genotypes. This study evaluated the relationship between molecular markers and root traits in 114 wild barley genotypes collected from the natural distributional range in western Iran. The genotypes were subjected to normal (90%–95% field capacity) and water-stress (50%–55% field capacity) conditions. Root, physiological and seedling traits were carefully measured, and the genotypes were analyzed using 35 molecular markers, including simple sequence repeats (SSRs) and expressed sequence tag-SSRs (EST-SSRs). Statistical association analyses were performed to assess the correlation between markers and root traits. The study revealed significant genetic diversity among the 114 wild barley genotypes, reflecting distinct environmental pressures in their regions of origin. Several molecular markers, especially BMAG0603 and GBM1126, consistently exhibited strong associations with desirable root traits, such as increased root length, root density, and seedling vigor under both normal and water-stressed conditions. These markers are valuable for marker-assisted selection (MAS) in breeding programs aimed at improving drought tolerance. Specific chromosomal regions critical for root trait development were identified, offering insights into the genetic control of drought tolerance in barley. The results highlight the importance of using molecular markers to enhance drought tolerance in barley. The identification of key markers associated with beneficial root traits offers a valuable resource for breeding programs focused on drought resilience. Further research should explore marker-trait associations under various stress conditions to optimize the genetic potential of wild barley for crop improvement strategies.

## Teaser

Hordeum spontaneum, the progenitor of cultivated barley, exhibits distinctive genetic traits that enhance drought tolerance. This study investigates the relationship between molecular markers and root traits in 114 wild barley genotypes under both normal and water-stress conditions. Utilizing SSR and EST-SSR markers, we identified key genetic regions associated with root length, root density, and seedling vigor, all of which are critical for drought resilience. Notably, the markers BMAG0603 and GBM1126 demonstrated strong associations with beneficial root traits, positioning them as valuable tools for marker-assisted selection (MAS) in breeding programs. These findings lay a foundation for enhancing barley's drought tolerance through molecular breeding, thereby promoting better adaptation to climate change and water-limited environments.

## Introduction

Wild barley [*Hordeum vulgare* subsp*. spontaneum* (Koch) Asch. & Graebn.] has emerged as a critical resource in the study of drought stress, largely due to its remarkable genetic diversity and adaptability to arid environments. Cultivated barley (*H. vulgare* L.) was selected from its wild progenitor only during the last 20,000 years (Weiss *et al.* 2008). Other species of subg. *Hordeum* such as *H. bulbosum* L. split 3.7 million years ago (mya), while *H. murinum* L. the third early diverging species of the clade separated ~ 8 mya (Brassac and Blattner 2015; Blattner 2018). Both wild and cultivated barley possess a similar diploid (2*n* = 14) *H* genome, around 4.5 Gb in size (Mascher *et al.* 2017; Liu *et al.* 2020; Zhang *et al.* 2023). They are primarily self-pollinating and fully inter fertile (ZoharyHopf and Weiss 2012), in contrast to other species of subg. *Hordeastrum*, which diverged 9.2 million years ago, exceeding the temporal limits for potential hybridization (Blattner 2009; Jakob *et al.* 2014). The process known as the domestication syndrome has produced changes in cultivated barely (Hammer 1984), but in the taxonomy of the genus *Hordeum*, the two are considered conspecific reflecting the current concept that the crop and its wild progenitor fall within the same clade. With this taxonomic distinction in mind, for the sake of simplicity hereafter we will refer to wild barley as *spontaneum*.

This species displays substantial phenotypic variation in drought tolerance, making it invaluable for breeding programs aimed at enhancing drought tolerance in cultivated barley and other crops (Najafipour *et al.* 2023). Moreover, wild barley serves as an effective model for exploring the physiological and molecular mechanisms underpinning drought response, knowledge that is crucial for the development of resilient agricultural systems in the face of climate change (Qiu *et al.* 2020; Pan *et al.* 2022). The genetic traits identified in wild barley have the potential to improve drought tolerance in domesticated varieties, thereby contributing to food security in regions vulnerable to water scarcity (Barati *et al.* 2018; Bakır *et al.* 2023). Despite the well-documented potential of wild barley, integrating these traits into commercial breeding programs remains a challenge, requiring further research to optimize the process (Barati *et al.* 2018; Najafipour *et al.* 2023).

Root systems play a pivotal role in drought tolerance by enhancing water uptake and improving plant resilience under water-limited conditions. Research indicates that deep root systems enable plants to access moisture from deeper soil layers, which is particularly vital during prolonged dry spells (Zhang *et al.* 2024). Additionally, root architecture, including traits such as root length and density, significantly influences a plant’s ability to withstand drought stress by expanding the soil volume explored for water (Wang *et al.* 2024). Certain root traits, such as the ability to form symbiotic relationships with mycorrhizal fungi, can further enhance drought tolerance by improving nutrient and water absorption (KalraGoel and Elias 2024). However, the effectiveness of these adaptations can vary among species and environmental conditions, indicating that root traits, while essential, should be considered alongside other physiological and morphological adaptations for comprehensive drought tolerance (Liu *et al.* 2023).

Research has shown that root systems play a critical role in drought tolerance. However, while wild barley genotypes generally exhibit superior drought tolerance compared to cultivated varieties (Barati *et al.* 2015, 2020), there are still gaps in understanding the genetic mechanisms that underlie root trait variability under drought conditions. Although previous studies have identified important root traits associated with drought tolerance in barley, further research is needed to fully elucidate the genetic and physiological basis of root trait variability across different environments (Sallam *et al.* 2019; Oyiga *et al.* 2020).

Association mapping analysis has emerged as a powerful tool for identifying genetic variants linked to specific traits in populations, with significant applications across various fields, including agriculture. For instance, its use in identifying quantitative trait loci (QTL) associated with yield traits in crops has demonstrated its potential to enhance breeding programs (Liu *et al.* 2024). This technique leverages linkage disequilibrium in diverse populations, eliminating the need for specific mapping populations (Breseghello and Sorrells 2006). Recent advancements in genotyping and computational methods, including mixed-effects models and machine learning algorithms like Random Forest, have enhanced the effectiveness of association mapping (Pino Del Carpio *et al.* 2011; Saba Rahim *et al.* 2018). However, population structure and kinship can confound results, necessitating statistical corrections.

In the context of barley, association mapping has been particularly valuable in pinpointing genes related to root traits, which are crucial for improving drought tolerance and overall adaptability to diverse environmental conditions (Ibrahim *et al.* 2020). However, the success of these efforts can be hindered by challenges such as environmental variability and population structure, which can complicate the mapping process and lead to spurious associations. To overcome these issues, integrating genomic data with phenotypic traits and employing careful design and analysis strategies are essential (Cao and Hou 2023; Gao *et al.* 2023). Despite these challenges, the integration of association mapping into barley breeding programs holds significant promise for advancing crop performance, particularly in optimizing root traits for enhanced resilience under stress conditions (Ahmad *et al.* 2019; Bangarwa *et al.* 2020).

The primary objective of this study is to enhance our understanding of the molecular and physiological mechanisms related to drought stress in wild barley, with a particular focus on the crucial role of roots in this process. In this context, association mapping using molecular markers is a powerful tool for identifying markers associated with important root traits and drought tolerance. This research aims to optimize the use of this technique and identify key molecular markers, which could pave the way for more effective and sustainable breeding strategies to enhance drought tolerance in barley and other crops.

## Materials and methods

### Material and experimental design

**Figure 1.**
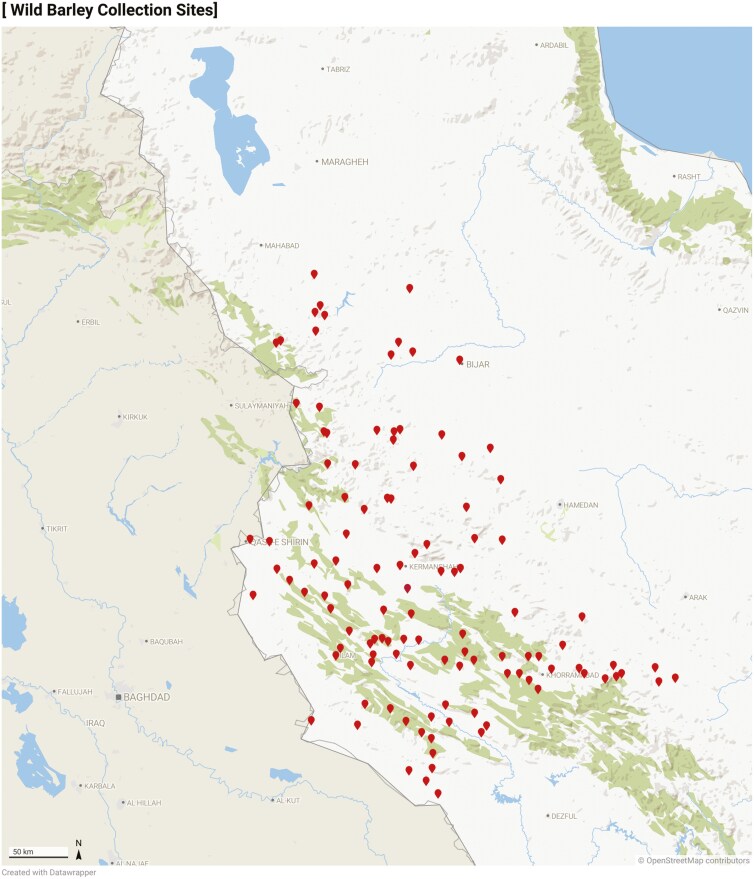
Distribution of the studied wild barley genotypes in western Iran.

This study was conducted on 114 genotypes of wild barley collected from the western provinces of Iran. The genotypes consisted of 29 from Kermanshah, 28 from Kurdistan, 28 from Ilam, and 29 from Lorestan (Figures 1). The formal identification of the genotypes was carried out by Ali A. Mehrabi. Additional information on the Ilam University gene bank code (IUGB), genotype numbers, collection locations/regions, and geographic coordinates can be found in the Supplementary Material Table S1. The genotypes in this study were collected based on the natural diversity found in different geographic regions. The selection of genotypes was guided by the ecological and natural variation present across these regions. No specific genetic screening or identification for traits of interest, such as drought tolerance or other genetic characteristics, was conducted during the selection process.

The experiment was conducted in the greenhouse of the Agricultural Research Center and Natural Resources of Kermanshah province in 2021, under both water stress and normal conditions. An augmented experiment was conducted using a completely randomized design with five replicates and nine control genotypes: 1 (Kermanshah- Mahidasht), 24 (Kermanshah- Sarpol-e Zahab), 35 (Kermanshah- Harsin), 10 (Ilam- Ilam), 51 (Ilam- Mehran), 74 (Kurdistan- Kamyaran), 34 (Kurdistan- Bijar), 104 (Lorestan- Kohdasht), and 113 (Lorestan- Aligudarz).

### Root evaluation

For the assessment of root traits during the seedling growth stage, 150 cm height and 12 cm diameter culture tubes were employed. The tubes were filled with sterilized sand to ensure a uniform growing medium. Seven seeds were initially planted in each tube, and post-germination, the seedlings were thinned to five per tube to maintain consistency in plant density. To meet the nutritional demands of the plants throughout the growth period, essential macro- and micronutrients were provided via Hoagland’s nutrient solution.

The culture tubes were placed in a greenhouse with natural light, where temperature conditions were carefully controlled, maintaining a daytime temperature of 25°C and a nighttime temperature of 15°C. Relative humidity was maintained at a level within the standard range, known to support optimal seedling growth in controlled greenhouse environments. Light intensity was monitored using a quantum sensor (LI-COR Biosciences) to ensure it was within the standard range, promoting healthy seedling growth.

After six weeks of growth, both root and aerial traits were measured. The six-week period is particularly suitable for species such as barley and other cereal crops, as it represents a stage where the roots have sufficiently developed and are measurable, yet remain under the influence of experimental conditions (such as moisture, light, and nutrition). During this phase, the roots exhibit distinctive growth characteristics, making it an optimal time to assess desirable traits and evaluate the plant’s response to stress factors. Root extraction was performed via destructive sampling, ensuring minimal disruption to the root system. The roots were carefully separated from the sand residue by washing them with gentle water pressure on a sloping surface to avoid mechanical damage. Following this, the aerial parts of the seedlings were separated from the roots for subsequent analysis.

To apply water stress, the field capacity (FC) of the soil and its moisture retention duration were initially calculated using the gravimetric method. For this, soil samples were taken and weighed before and after drying to determine the soil’s FC. Stress was applied at 50%–55% FC of the soil’s field capacity. Drought stress was introduced at the 14-day seedling stage after the initial growth and establishment of seedlings. Stress conditions were managed at two FC levels: normal (FC = 90%–95%) and stressed (FC = 50%–55%), with the stress period lasting four weeks.

For the measurement of field capacity, a Sartorius balance with a precision of 0.001 g (Sartorius, Germany) was used to determine the weight difference in the soil samples. The field capacity was calculated by measuring the soil’s weight before and after drying. Seedling fresh weight, root fresh weight, seedling dry weight, and root dry weight were measured in grams using a balance with an accuracy of 0.001 g. Root length and seedling length were measured in centimeters using a ruler.

Root traits such as root area (2 × SQRT (root volume × 3.14 × root length) (Shaban *et al.* 2012), root diameter (SQRT [(4 × root fresh weight) / (root length × 3.14)]) (Schenk and Barber 1979; Hajabbasi 2001), specific root length (root length / root dry weight) (HuangTaylor and McMichael 1991; Mahanta *et al.* 2014), root length density (root length / soil volume) (Mandal *et al.* 2003; Mahanta *et al.* 2014), root specific mass (root dry weight / soil volume) (Hajabbasi 2001; Hasanabadi *et al.* 2010), root tissue density (root dry weight × root volume) (Paula and Pausas 2011), root mass density (root fresh weight / soil volume) (Hajabbasi 2001), Root fineness (root length / root volume) (Hajabbasi 2001) and root surface area density (root length × root diameter × 3.14) (Ali *et al.* 2018). were calculated using the fresh weight, dry weight, and root length data, along with soil volume. Root volume was determined using a graduated cylinder: the change in water volume after immersing the plant roots was recorded.

The Lichtenthaler and Welburn (1983) method was used to measure chlorophyll and carotenoid content. Twenty five milligrams of leaves were powdered in a Chinese mortar with liquid nitrogen and then completely homogenized with 2 ml of 96% ethanol in the dark (Lichtenthaler and Wellburn 1983). To homogenize the obtained solution, the tubes were shaken and centrifuged for 10 minutes at 10,000 rpm at 4°C. The sample supernatants were then poured into a plate and read with an ELISA device (BioTek PowerWave XS, USA) at wavelengths 663, 646, and 470 nm. The amounts of chlorophyll a, chlorophyll b, total chlorophyll, and carotenoids were calculated with the following formulas:


$$\begin{array}{l} Chl\;a\;=\;12.21\;\left(A_{663}\right)\;--\;2.81\;(A_{646}) \end{array}$$

$$\begin{array}{l} Chl\;b\;=\;20.13\;\left(A_{646}\right)\;--\;5.1\;(A_{663}) \end{array}$$

$$\begin{array}{l} Chl\;T\;=\;Chl\;a\;+\;Chl\;b \end{array}$$

$$\begin{array}{l} Car\;=\;(1000\;A_{470}--\;3.27\;\left[Chl\;a\right]\;--\;104\;\left[Chl\;b\right]/227 \end{array}$$

An analysis of variance (ANOVA) was performed on the genotypic groups using SPSS software. Cluster analysis of the traits was conducted using Ward’s method, facilitated by ClustVis software (Metsalu and Vilo 2015).

### DNA extraction and genotyping

DNA extraction was performed on each population using a modified CTAB method (TorresWeeden and Martin 1993). The quality and quantity of the extracted DNA were assessed through 0.8% agarose gel electrophoresis and spectrophotometric analysis. Polymerase Chain Reaction (PCR) was conducted in a 20 μl reaction volume, containing 50 ng template DNA, 2 mM MgCl_2_, 0.05 mM of each dNTP, 0.2 μM primers, one unit of Taq DNA Polymerase, and 1X reaction buffer (Cinnagen, Iran). The thermal cycling protocol consisted of an initial denaturation at 95°C for 5 minutes, followed by 35 cycles of 30 seconds at 95°C, 30 seconds at a primer-specific annealing temperature (ranging from 52 to 60°C), and 60 seconds at 72°C, with a final extension at 72°C for 5 minutes. DNA bands were visualized using a 4% agarose gel with 1X TBE buffer and the Quantum ST4 Gel Documentation System. Finally, the obtained data were evaluated using software tools: Darwin 6 for distance matrix computation and cluster analysis.

### Genetic diversity snalysis

Using 35 molecular primers (sequences provided in the Supplementary Material Table S2); (Ramsay *et al.* 2000; Ramsay *et al.* 2004; GrainGenes: https://wheat.pw.usda.gov/GG3/), and based on the marker information obtained from the GrainGenes database, the genotypes were systematically categorized into distinct subpopulations. Mixed genotypes were identified employing the Bayesian approach in Structure 2.3.4 software (PritchardStephens and Donnelly 2000). The initial values of *K* (representing the number of hypothetical subpopulations) were tested between 1 and 10. To ensure accuracy, each *K* value was repeated five times. The admixture model with allele frequency independence was applied, utilizing 10 000 Burn-in and 100 000 repetitions (Markov Chain Monte Carlo, MCMC) to derive the maximum likelihood curve. The Structure software calculates a matrix called *Q* for each *K* value, estimating the probability coefficients of each genotype’s membership in each subpopulation. A genotype was assigned to a cluster if its membership percentage was greater than or equal to 0.7; otherwise, it was classified as a hybrid genotype (Mixed) (Spataro *et al.* 2011).

The estimated number of subpopulations (*K*) was determined using two methods. The first method relied on the Structure selection criterion, LnP(D), which evaluates the posterior probability of the data across various *K* values. The second method employed the Δ*K* statistic, as proposed by Evanno *et al.* (2005), which identifies the value of *K* with the highest probability based on the LnP(D) function (EvannoRegnaut and Goudet 2005).

### Association analysis

To identify markers significantly associated with the evaluated traits, a mixed linear model (MLM) approach was utilized. Marker-Trait Association (MTA) analysis was performed using TASSEL 3 software. Prior to MTA analysis, several data quality control measures were implemented: low-quality alleles and genotypes with high missing data were excluded, and markers with a Minor Allele Frequency (MAF) less than 5% were removed to avoid unreliable associations. The genotypic data were tested for Hardy–Weinberg Equilibrium (HWE) using TASSEL, and linkage disequilibrium (LD) between markers was calculated. To account for population stratification, the Q-matrix was extracted using STRUCTURE software and incorporated into the MTA analysis. Additionally, the Kinship (K-matrix) was computed using TASSEL and included as a covariate in the association model to account for genotype relatedness. The MLM in TASSEL was employed to identify MTAs, considering the *Q* + *K* matrix to minimize false associations between trait markers.

### Identification of marker positions

The positions of the markers were identified using the data provided in the Supplementary File (available in Supplementary Table S2) and retrieved from the GrainGenes database (https://wheat.pw.usda.gov/GG3/), where the positions are specified in centimorgan (cM). This approach was selected due to the higher accuracy of cM-based maps for Marker-Trait Association (MTA) studies. The chromosome map was generated using the ggplot2 software package in the R programming environment. This powerful tool allows for the construction of precise and visually interpretable genetic maps in cM units, facilitating the identification and localization of molecular markers across chromosomes. This approach ensures high accuracy and flexibility in visualizing genetic relationships, making it particularly suitable for advanced genetic analysis and trait association studies.

## Results

### Root and shoot traits evaluation

The results of variance analysis of adjusted treatments in the form of factorial design showed that there was a significant difference between different genotypes of wild barley except for root diameter. Also, in the drought stress levels, except for root diameter and root area traits, there is a significant difference at the level of 1% for all traits (Table 1), also the interaction effect of genotype in water stress is non-significant for root diameter trait, for the traits seedling length, root dry weight, root surface area density and chlorophyll a were significant at the 5% level, and significant differences were observed for other traits at the 1% level (Table 1).

**Table 1.** Results of variance analysis of adjusted treatments for wild barley root and seedling traits.

S.O.V	Df	Ms									
		Root length	Seedling length	Root fresh weight	Seedling fresh weight	Seedling dry weight	Root volume	Root dry weight	Root fineness	Root diameter	Specific root length
Repetition	4	1.761	1.317	0.010	0.111	0.007	0.00000421	0.0001	26.819	0.0000041	308.242
Stress	1	7670.411^**^	860.009^**^	8.293**	13.504^**^	1.571^**^	0.000031^**^	0.297^**^	127241.581^**^	0.0000048^ns^	6853922.124^**^
Genotypes	113	118.610^**^	14.089^**^	0.128^**^	0.416^**^	0.052^**^	0.00000291^**^	0.003^**^	4432.639^**^	0.0000101^ns^	121338.401^**^
Genotypes × Stress	113	57.652^**^	6.416^*^	0.072^**^	0.134^**^	0.015^**^	0.00000241^*^	0.002^*^	3470.294^**^	0.0000096^ns^	91512.179^**^
Error	72	9.205	4.011	.007	.025	.003	0.00000144	0.0001	143.316	0.0000070	2213.538
S.O.V	Df	Ms									
		Root length density	Root specific mass	Root tissue density	Root mass density	Root surface area density	Root area	Chlorophyll a	Chlorophyll b	Carotenoid	Total chlorophyll
Repetition	4	0.0000007	0.000000006	33.496	0.0000004	0.849^**^	0.058^*^	.393	.208	.478	.776
Stress	1	0.00269**	0.000000104**	12271.423**	0.0002914**	4.028**	0.066ns	83.027**	59.563**	525.036**	283.237**
Genotypes	113	0.000041**	0.000000130**	252.959**	0.0000045**	0.241**	0.179**	.625**	0.447**	2.959**	1.8128**
Genotypes × Stress	113	0.0000201**	0.000000783**	313.956**	0.0000025**	0.163*	0.116**	.436**	0.384**	2.489**	1.375**
Error	72	0.0000033	0.00000000004	50.341	0.000000003	.109	.019	.212	0.117	0.522	.409

-Sources of variation (S.O.V), mean of squares (MS), degree of freedom (df).

* and ** are significant at the 5% and 1% probability level respectively, and ^ns^ are not significant.

Tables 2 and 3 show the results of the LSD test for different traits measured respectively in the normal conditions (FC = 90%–95%) and water stress (FC = 50%–55%) at the probability level of 5% and 1% they show. In water stress conditions, a high coefficient of variation was assigned to root volume, root dry weight, root fineness, root diameter, specific root length, root specific mass, root tissue density, root mass density, and root area. The minimum coefficient of variation also belonged to seedling length.

**Table 2.** Comparison of mean genotypes of wild barley in the normal condition for root and seedling traits.

Genotype	Root length (cm)	Seedling length(cm)	Root fresh weight (gr)	Seedling fresh weight (gr)	Seedling dry weight (gr)	Root volume (cm^3^)	Root dry weight (gr)	Root fineness (cm/gr)	Root diameter (cm)	Specific root length (cm/gr)
LSD 5%	3.881	2.562	0.107	0.202	0.070	0.001537	0.012790	15.312	0.003385	60.176
LSD 1%	5.183	3.421	0.143	0.270	0.094	0.002052	0.017084	20.452	0.004521	80.375
Minimum	50	30	0.387	0.900	0.508	0.000735	0.052000	28.345	0.003072	131.579
Maximum	95	46	1.788	4.301	1.892	0.010190	0.390000	186.047	0.016919	1290.323
mean	68.789	38.509	0.852	2.376	1.220	0.004065	0.136430	93.422	0.007586	626.296
Variation range	45.000	16.000	1.401	3.401	1.384	0.009455	0.338000	157.702	0.013847	1158.744
Standard deviation	7.563	3.078	0.363	0.558	0.210	0.001825	0.071606	34.064	0.002584	271.954
Standard error	0.066	0.027	0.003	0.005	0.002	0.000016	0.000628	0.299	0.000023	2.386
Coefficient of variation	10.993	7.992	42.573	23.489	17.192	44.886	52.485	36.462	34.062	43.422
Inferior genotype	92, 108, 82, 96, 87, 111, 70 and 112	2, 35, 39, 44, 45, 46 and 101	113, 114, 111, 112, 110, 92, 109, 83, 48 and 11	112, 11, 77, 83, 87, 88 and 45	112, 77, 110, 106, 84 and 94	113, 110, 109, 28, 22, 88, 89, 24, 11, 48 and 81	91, 113, 83, 77, 112, 114, 94, 111, 101 and 110	108, 63, 73, 107, 68, 66 and 82	11, 113, 62, 15, 9, 13, 114, 3, 4 and 64	108, 79, 42, 69, 21, 70, 89 and 34
Superior genotype	11, 15, 9, 13, 62 and 4	91, 90, 93, 6, 42, 56, 60, 68, 69, 77, 79 and 92	108, 63, 73, 68, 107, 66, 102, 6, 95, 34, 10, and 78	91, 93, 40, 90, 26, 17, 18 and 42	91, 4, 9, 16, 40, 56, 64, 69 and 30	20, 63, 68, 108, 73, 6, 107, 18, 83 and 102	108, 21, 6, 79, 5, 42, 69, 7, 12, 27 and 37	11, 113, 114, 62, 111, 110, 112, 83, 15 and 48	108, 63, 107, 82, 73, 66, 68, 95 and 34	91, 11, 62, 83, 113, 77, 103, 100 and 15

Genotype	Root length density (cm^3^)	Root specific mass (gr/cm^3^)	Root tissue density (gr/cm^3^)	Root mass density (gr/cm^3^)	Root surface area density (cm²/cm³)	Root area (cm^2^)	Chlorophyll a (mg g^-1^ fw)	Chlorophyll b (mg g^-1^ fw)	Carotenoids (mg g^-1^ fw)	Total chlorophyll (mg g^-1^ fw)
LSD 5%	0.00230588	0.00000805	9.075	0.00006402	0.422	0.176	0.589	0.437	0.924	0.818
LSD 1%	0.00307988	0.00001075	12.121	0.00008550	0.564	0.235	0.787	0.584	1.234	1.093
Minimum	0.02963841	0.00003082	13.022	0.00022940	0.657	0.346	5.222	2.556	11.965	8.370
Maximum	0.05631298	0.00023118	97.551	0.00105957	2.674	1.486	9.667	5.464	20.621	14.898
Mean	0.04077621	0.00008087	35.800	0.00050496	1.634	0.913	6.717	4.243	15.672	10.959
Variation range	0.02667457	0.00020036	84.529	0.00083017	2.017	1.139	4.445	2.908	8.656	6.528
Standard deviation	0.00448292	0.00004245	17.761	0.00021498	0.552	0.227	0.699	0.637	1.481	1.225
Standard error	0.00003932	0.00000037	0.156	0.00000189	0.005	0.002	0.006	0.006	0.013	0.011
Coefficient of variation	10.993	52.485	49.611	42.573	33.777	24.851	10.406	15.003	9.452	11.174
Inferior genotype	92, 108, 82, 96, 87, 111, 70 and 112	91, 113, 83, 77, 112, 114, 94, 111, 101 and 110	83, 20, 63, 101, 67, 77, 68, 73, 94 and 100	113, 114, 111, 112, 110, 92, 109, 83, 48 and 11	113, 114, 111, 112, 110, 92, 109, 83, 48 and 11	92, 81, 88, 89, 85, 87, 86 and 93	92, 77, 73, 64, 83, 78, 26 and 87	48, 44, 2, 61, 62, 12, 92, 57 and 77	108, 14, 48, 44, 1, 92, 61, 62 and 2	92, 77, 73, 2, 48, 44, 78, 83, 12 and 87
Superior genotype	11, 15, 9, 13, 62 and 4	108, 21, 6, 79, 5, 42, 69, 7, 12, 27 and 37	89, 27, 12, 22, 24, 21, 28, 42, 71, 49, 70 and 50	108, 63, 73, 68, 107, 66, 102, 6 and 95	108, 63, 73, 68, 107, 66, 102, 6 and 95	20, 63, 6, 13, 15, 68, 18, 10, and 102	65, 114, 110, 34, 6, 32, 41, 16, 109 and 40	6, 34, 32, 16, 41, 40, 15 and 39	20, 34, 32, 41, 13, 40, 39 and 73	6, 34, 32, 16, 41, 40, 15 and 39

**Table 3.** Comparison of mean genotypes of wild barley in the water stress condition for root and seedling traits.

Genotype	Root length (cm)	Seedling length(cm)	Root fresh weight (gr)	Seedling fresh weight (gr)	Seedling dry weight (gr)	Root volume (cm^3^)	Root dry weight (gr)	Root fineness (cm/gr)	Root diameter (cm)	Specific root length (cm/gr)
LSD 5%	3.881	2.562	0.107	0.202	0.070	0.002	0.013	15.312	0.003385	60.176
LSD 1%	5.183	3.421	0.143	0.270	0.094	0.002	0.017	20.452	0.004521	80.375
Minimum	22.000	25.000	0.100	0.765	0.315	0.001	0.022	30.726	0.002686	259.459
Maximum	83.000	41.000	1.920	3.653	1.775	0.008	0.185	750.000	0.024498	3409.091
Mean	57.667	34.675	0.485	1.904	1.060	0.003	0.066	139.079	0.007806	963.281
Variation range	61.000	16.000	1.820	2.888	1.460	0.007	0.163	719.274	0.021813	3149.631
Standard deviation	9.999	3.107	0.221	0.456	0.142	0.001	0.024	79.498	0.002408	351.386
Standard error	0.088	0.027	0.002	0.004	0.001	0.000	0.000	0.697	0.000021	3.082
Coefficient of variation	17.339	8.961	45.590	23.936	13.365	36.628	36.504	57.160	30.845	36.478
Inferior genotype	93, 101, 102, 92, 14, 81, 83, 103, 112 and 111	11, 35, 50, 101, 12, 36, 38, 97, 24, 65, 96 and 102	111, 105, 104, 62, 110, 84, 101, 89, 73, 100 and 107	111, 48, 112, 114, 113, 108, 89, 101 and 84	112, 99, 113, 48, 102, 54, 98, 94 and 85	111, 105, 104, 110, 84, 101, 99 and 94	111, 105, 104, 110, 84 and 101	19, 15, 67, 86, 16, 56, 14 and 9	105, 62, 104, 110, 113, 107, 89, 84, 73 and 52	14, 15, 16, 13, 19, 5, 7, 90, 9 and 4
Superior genotype	105, 91, 18, 51, 10, 64, 9, 27, 29, 32, 37 and 49	42, 92, 56, 68, 92, 18, 23, 64 and 105	19, 9, 15, 16, 64, 56, 86, 67, 12, 66 and 3	91, 93, 16, 9, 37, 3, 64, 56 and 4	91, 12, 64, 9, 16, 69, 22, 37 and 56	15, 16, 6, 13, 9, 5, 18, 14 and 12	15, 16, 6, 13, 9, 5 and 18	105, 62, 104, 110, 107, 113, 111, 89, 84 and 73	34, 50, 36, 43, 48, 39, 33, 46, 46, 42, 49, 44 and 32	105, 104, 111, 110, 91, 84, 62, 94 and 57

Genotype	Root length density (cm^3^)	Root specific mass (gr/cm^3^)	Root tissue density (gr/cm^3^)	Root mass density (gr/cm^3^)	Root surface area density (cm²/cm³)	Root area (cm^2^)	Chlorophyll a (mg g^-1^ fw)	Chlorophyll b (mg g^-1^ fw)	Carotenoids (mg g^-1^ fw)	Total chlorophyll (mg g^-1^ fw)
LSD 5%	0.00231	0.00000805	9.075	0.00006402	0.422	0.176	0.589	0.437	0.924	0.818
LSD 1%	0.00308	0.00001075	12.121	0.00008550	0.564	0.235	0.787	0.584	1.234	1.093
Minimum	0.01304	0.00001304	8.829	0.00005928	0.632	0.419	3.168	1.305	6.312	4.766
Maximum	0.04920	0.00010966	78.409	0.00113811	2.771	2.535	6.066	3.770	13.396	9.431
Mean	0.03418	0.00003896	20.894	0.00028746	1.365	0.963	4.453	2.605	10.213	7.058
Variation range	0.03616	0.00009662	69.580	0.00107884	2.139	2.116	2.897	2.465	7.084	4.666
Standard deviation	0.00593	0.00001422	10.340	0.00013105	0.277	0.483	0.551	0.508	1.391	0.997
Standard error	0.00005	0.00000012	0.091	0.00000115	0.002	0.004	0.005	0.004	0.012	0.009
Coefficient of variation	17.339	36.504	49.487	45.590	20.304	50.208	12.371	19.505	13.615	14.121
Inferior genotype	93, 101, 102, 92, 14, 81, 83, 103, 112, and 111	111, 105, 104, 110, 84, 101, 99 and 94	29, 86, 28, 110, 26, 71, 29, 27, 99, and 111	111, 105, 104, 62, 110, 84, 101, 89, 73, 100 and 107	111, 105, 104, 62, 110, 84, 101, 89, and 73	89, 81, 73, 84, 92, 100, 62, 101, 83, and 112	62, 57, 73, 20, 19, 78, 3, 21, 99, and 102	99, 102, 62, 57, 98, 52, and 35	62, 111, 57, 73, 99, 102, 78, 98, and 94	62, 57, 99, 20, 73, 102, 19, 98, 52, 3, 35, 94, and 21
Superior genotype	105, 91, 18, 51, 10, 64, 9, 27, 29, 32, 37, and 49	15, 16, 6, 13, 9, 5, 18, 14, and 12	39, 44, 40, 79, 38, 51, 50, 7, 43, 41, and 20	19, 9, 15, 16, 64, 56, 86, 67, 12, 66, and 3	50, 34, 49, 51, 32, 43, 39, 42, and 36	91, 29, 19, 30, 9, 16, 3, 26, 18. and 12	12, 56, 9, 16, 91, 29, 5, 109, 64, 110, and 30	109, 91, 64, 90, 110, 107, 56, 67, 105, 108, and 113	91, 90, 56, 107, 105, 55, 97, 50, and 103	109, 91, 56, 64, 90, 12, 110, 107, and 67

The mean root length and root length density were equal to 68.78 and 0.407, genotypes 11, 15, 9, 13, 62, and 4 had the lowest root length and root length density in the normal condition, and genotypes 92, 108, 82, 96, 87, 111, 70, and 112 had the highest root length and root length density. The variation range of seedling length was 16 with a mean of 38.50, genotypes 91, 90, 93, 6, 42, 56, 60, 68, 69, 77, 79, and 92 had the lowest seedling length in the normal condition, and in the genotypes 2, 35, 39, 44, 45, 46 and 101 had the highest seedling length. The mean root fresh weight was equal to 0.85, in the normal condition, genotypes 108, 63, 73, 68, 107, 66, 102, 6, 95, 34, 10, and 78 had the lowest root fresh weight, and genotypes 113, 114, 111, 112, 110, 92, 109, 83, 48 and 11 had the highest root fresh weight. In the normal condition, the seedling fresh weight had a mean of 2.37, genotypes 91, 93, 40, 90, 26, 17, 18, and 42 had the lowest seedling fresh weight in the normal condition, and genotypes 112, 11, 77, 83, 87, 88, and 45 had the highest seedling fresh weight. The mean seedling dry weight was 1.22 with a standard deviation of 0.21. Genotypes 91, 4, 9, 16, 40, 56, 64, 69, and 30 have the lowest seedling dry weight in normal condition, and genotypes 112, 77, 110, 106, 84, and 94 have the highest seedling weight dry weight was observed. Genotypes 20, 63, 68, 108, 73, 6, 107, 18, 83, and 102 have the lowest root volume in normal condition, and genotypes 113, 110, 109, 28, 22, 88, 89, 24, 11, 48 and 81 had the highest root volume. Root dry weight had a mean of 0.136 with a standard deviation of 0.071. Genotypes 108, 21, 6, 79, 5, 42, 69, 7, 12, 27, and 37 have the lowest root dry weight in normal condition, and genotypes 91, 113, 83, 77, 112, 114, 94, 111, 101 and 110 had the highest root dry weight. The variation range in root fineness was equal to 157.7 with a mean of 93.42, genotypes 11, 113, 114, 62, 111, 110, 112, 83, 15, and 48 had the lowest root fineness in the normal condition, and genotypes 108, 63, 73, 107, 68, 66 and 82 had the most root fineness. The mean root diameter was equal to 0.007 with a variation range of 0.013. genotypes 108, 63, 107, 82, 73, 66, 68, 95, and 34 have the lowest root diameter in normal condition, and in genotypes 11, 113, 62, 15, 9, 13, 114, 3, 4, and 64 the highest root diameter was observed. The mean specific root length was 29.626, genotypes 91, 11, 62, 83, 113, 77, 103, 100, and 15 had the lowest specific root length in the normal condition, and genotypes 108, 79, 42, 69, 21, 70, 89 and 34 had the highest specific root length in the normal condition. Genotypes 108, 21, 6, 79, 5, 42, 69, 7, 12, 27, and 37 have the lowest root specific mass in the normal condition, and genotypes 91, 113, 83, 77, 112, 114, 94, 111, 101, and 110 have the highest root specific mass in the normal condition. The variation range of root tissue density changes was 84.52 with a mean of 35.8, genotypes 89, 27, 12, 22, 24, 21, 28, 42, 71, 49, 70, and 50 had the lowest root tissue density in the normal condition and the highest root tissue density was observed in genotypes 83, 20, 63, 101, 67, 77, 68, 73, 94 and 100. The mean root mass density was equal to 0.00005, and the mean and standard deviation of root surface area density were equal to 1.63 and 0.552, respectively, in genotypes 108, 63, 73, 68, 107, 66, 102, 6, and 95, also the lowest root mass density and root area density in the normal condition and genotypes 113, 114, 111, 112, 110, 92, 109, 83, 48, and 11 have the highest root mass density and root surface area density. The mean and variation range of root area changes were 0.91 and 1.13 respectively, genotypes 20, 63, 6, 13, 15, 68, 18, 10, and 102 had the lowest root area in the normal condition, and genotypes 92, 81, 88, 89, 85, 87, 86 and 93 had the highest root area. Genotypes 65, 114, 110, 34, 6, 32, 41, 16, 109, and 40 have the lowest chlorophyll a in normal condition, and genotypes 92, 77, 73, 64, 83, 78, 26, and 87 had the most chlorophyll a. The variation range of total chlorophyll changes was 6.52, genotypes 6, 34, 32, 16, 41, 40, 15, and 39 had the lowest chlorophyll b and total chlorophyll in the normal condition and genotypes 48, 44, 2, 61, 62, 12, 92, 57 and 77 had the highest chlorophyll b and genotypes 92, 77, 73, 2, 48, 44, 78, 83, 12 and 87 had the highest total chlorophyll. The mean carotenoid was 15.67 with a standard deviation of 1.48, genotypes 20, 34, 32, 41, 13, 40, 39, and 73 had the lowest carotenoid in the normal condition, and genotypes 108, 14, 48, 44, 1, 92, 61, 62 and 2 had the most carotenoids.

In the normal condition, the highest coefficient of variation in root fresh weight, seedling fresh weight, root volume, root dry weight, root fineness, root diameter, specific root length, root specific mass, root tissue density, root mass density, root surface area density, and root area was observed. The lowest coefficient of variation was also found in seedling length.

The mean root length and root length density under water stress conditions were equal to 66.57 and 0.341, genotypes 105, 91, 18, 51, 10, 64, 9, 27, 29, 32, 37, and 49 had the lowest root length and root length density, and the highest amount was observed in genotypes 93, 101, 102, 92, 14, 81, 83, 103, 112 and 111. The variation range of seedling length was 16 with a mean of 34.67, genotypes 42, 92, 56, 68, 92, 18, 23, 64, and 105 had the lowest seedling length in the water stress condition, and genotypes 11, 35, 50, 101, 12, 36, 38, 97, 24, 65, 96 and 102 had the highest seedling length. The mean root fresh weight and root mass density were equal to 0.485 and 0.0002, genotypes 19, 9, 15, 16, 64, 56, 86, 67, 12, 66, and 3 had the lowest root fresh weight and root mass. density in the water stress condition and genotypes 111, 105, 104, 62, 110, 84, 101, 89, 73, 100, and 107 had the highest values. In the water stress, seedling fresh weight had a mean of 1.90, and genotypes 91, 93, 16, 9, 37, 3, 64, 56, and 4 had the lowest seedling fresh weight, and genotypes 111, 48, 112, 114, 113, 108, 89, 101 and 84 were observed the most. The mean seedling dry weight was 1.06 with a standard deviation of 0.142. Genotypes 91, 12, 64, 9, 16, 69, 22, 37, and 56 have the lowest seedling dry weight in the water stress condition, and genotypes 112, 99, 113, 48, 102, 54, 98, 94, and 85 had the highest seedling dry weight. Genotypes 15, 16, 6, 13, 9, 5, 18, 14, and 12 have the lowest root volume and root specific mass in the water stress condition, and genotypes 111, 105, 104, 110, 84, 101, 99, and 94 had the highest amount. Root dry weight had a mean of 0.066 with a standard deviation of 0.024. Genotypes 15, 16, 6, 13, 9, 5, and 18 had the lowest root dry weight in the water stress condition and genotypes 111, 105, 104, 110, 84, and 101 had the highest root dry weight. The variation range of changes in root fineness was equal to 719.27 with a mean of 139.079, genotypes 105, 62, 104, 110, 107, 113, 111, 89, 84, and 73 had the lowest root fineness in the water stress condition and genotypes 19, 15, 67, 86, 16, 56, 14 and 9 have the most root fineness.

The mean root diameter was equal to 0.007 with a variation range of 0.021. Genotypes 34, 50, 36, 43, 48, 39, 33, 46, 46, 42, 49, 44, and 32 have the lowest root diameter in the water stress, and genotypes 105, 62, 104, 110, 113, 107, 89, 84, 73 and 52, the highest root diameter was observed. The mean specific root length was 963.28, the lowest specific root length in the water stress was genotypes 105, 104, 111, 110, 91, 84, 62, 94, and 57, and the highest was genotypes 14, 15, they had 16, 13, 19, 5, 7, 90, 9, and 4. The variation range of root tissue density changes was 69.58 with a mean of 88920, genotypes 39, 44, 40, 79, 38, 51, 50, 7, 43, 41, and 20 had the lowest root tissue density in the water stress and genotype 29, 86, 28, 110, 26, 71, 29, 27, 99, and 111 had the highest root tissue density. The mean and standard deviation of root surface area density were 1.36 and 0.277 respectively, genotypes 50, 34, 49, 51, 32, 43, 39, 42, and 36 had the lowest root surface area density in the water stress, and the highest root surface area density was observed in genotypes 111, 105, 104, 62, 110, 84, 101, 89, and 73. The mean and variation range of root area changes were 0.963 and 2.11 respectively, genotypes 91, 29, 19, 30, 9, 16, 3, 26, 18, and 12 had the lowest root area in water stress, and genotypes 89, 81, 73, 84, 92, 100, 62, 101, 83, and 112 had the most root area. In the water stress condition, genotypes 12, 56, 9, 16, 91, 29, 5, 109, 64, 110, and 30 have the lowest chlorophyll a and genotypes 62, 57, 73, 20, 19, 78, 3, 21, 99, and 102 had the most chlorophyll a. Genotypes 109, 91, 64, 90, 110, 107, 56, 67, 105, 108, and 113 have the lowest chlorophyll b in the water stress, and genotypes 99, 102, 62, 57, 98, 52, and 35 had the most chlorophyll b. The mean carotenoid was 10.21 with a standard deviation of 1.39, in the water stress genotypes 91, 90, 56, 107, 105, 55, 97, 50, and 103 had the lowest carotenoid and genotypes 62, 111, 57, 73, 99, 102, 78, 98, and 94 had the highest amount. The variation range of total chlorophyll changes was 4.66, genotypes 109, 91, 56, 64, 90, 12, 110, 107, and 67 had the lowest total chlorophyll in the water stress, and genotypes 62, 57, 99, 20, 73, 102, 19, 98, 52, 3, 35, 94 and 21 were observed the most total chlorophyll.

The groupings and clustering results were analyzed in more detail to better understand the relationships among the genotypes under both water stress and normal conditions.

**Figure 2.**
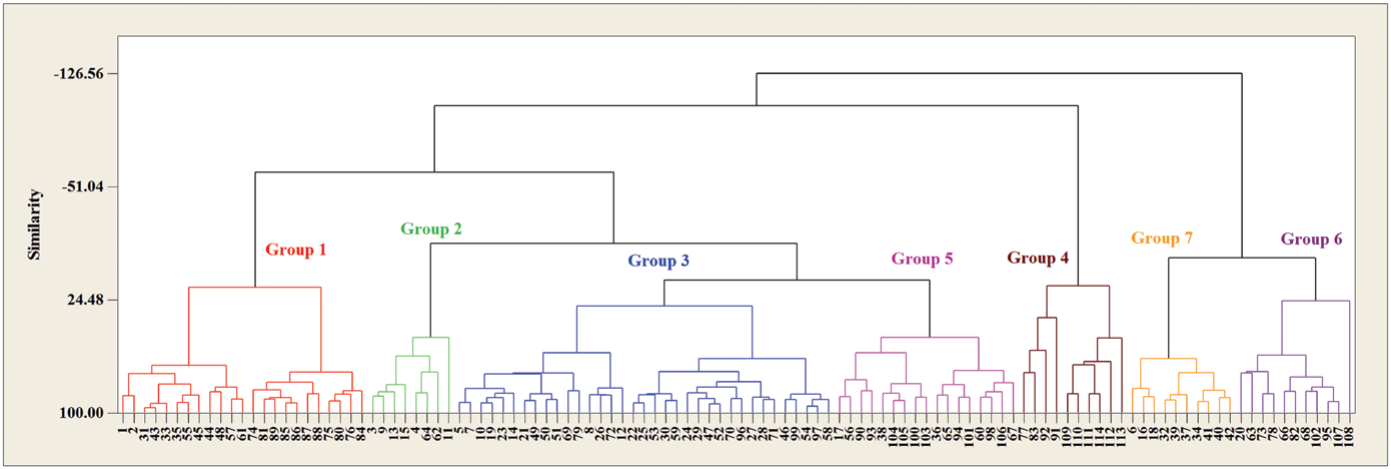
Dendrogram resulting from cluster analysis by Ward’s method under water stress conditions for wild barley root and seedling traits.

**Figure 3.**
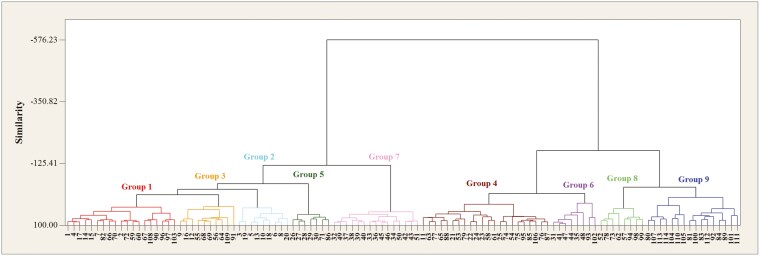
Dendrogram resulting from cluster analysis by Ward’s method in normal conditions for wild barley root and seedling traits.

Cluster analysis, using the WARD method, revealed a clear distinction between the genotypes in both stress and normal conditions (Figures 2 and 3). Under water stress, the genotypes were grouped into seven distinct clusters. Group 1 consisted of 23 genotypes, Group 2 included 8 genotypes, Group 3 included 35 genotypes, Group 4 contained 10 genotypes, Group 5 included 17 genotypes, Group 6 included 11 genotypes, and Group 7 included 10 genotypes.

Notably, Group 4, including genotypes 77, 83, 92, 91, 109, 110, 111, 112, 113, and 114, was characterized by having the most favorable root and plant characteristics, indicating their potential resilience under stress. Conversely, Group 6, which included genotypes 20, 63, 73, 78, 66, 82, 68, 102, 95, 107, and 108, was composed of some of the weakest genotypes based on the mean comparison table, highlighting their susceptibility to water stress.

In normal conditions, the genotypes were placed in nine distinct groups. Group 1 included 19 genotypes, Group 2 included 9 genotypes, Group 3 included 10 genotypes, Group 4 included 22 genotypes, Group 5 included 7 genotypes, Group 6 included 8 genotypes, Group 7 included 15 genotypes, Group 8 included 8 genotypes, and Group 9 included 16 genotypes.

The genotypes in Group 9, including genotypes 80, 107, 113, 114, 104, 110, 105, 81, 100, 83, 112, 92, 84, 89, 101, and 111, exhibited the highest levels of root and seedling characteristics, reflecting their superior performance under normal conditions. On the other hand, Group 3, which included genotypes 9, 16, 12, 55, 68, 69, 56, 64, 109, and 91, consisted of some of the weakest genotypes, as evidenced by the comparison table.

To provide a clearer understanding, the relationship between Figures 2 and 3 has been analyzed. Figures 2 and 3 represent clustering results under water stress and normal conditions, respectively. A comparison of the genotypic distribution across these two figures shows how the performance of the genotypes shifts between the two conditions.

In water stress, some genotypes that were placed in higher-performing groups under normal conditions (such as Group 9 in Figure 3) shifted to lower-performing groups (such as Group 6 in Figure 2), indicating the detrimental effects of stress on these genotypes. Conversely, some genotypes remained relatively stable or showed slight changes in their performance, highlighting the differential response of genotypes to water stress.

This analysis not only provides a more detailed view of the genotype performance under different environmental conditions but also reveals potential candidates for breeding programs targeting water stress resilience.

### Genetic diversity and population differentiation

The molecular diversity of the genotypes was assessed utilizing a set of 35 markers, comprising both SSR and EST-SSR types (electrophoresis gel images for some markers are presented in the Supplementary Material Figure S1). The alleles detected ranged from 2 to 4 per primer. Notably, the highest allele counts were obtained from the primers BMAG0189, HVLTPPB, SCSSR04163, and GBM1110. In contrast, primers such as BMAC0154, BMAG0508A, BMAG0603, EBMAC0970, EBMATC0054, BMAG0381, EBMAC0521, EBMAC0674, GBM1459, GBM1176, SCSSR18076, and GBM1212 showed the lowest frequencies of alleles. In total, the primers produced 97 distinct alleles, with an average of 2.77 alleles per marker. The polymorphic information content (PIC) for the primers ranged from 50% to 100%, demonstrating significant variation across the genotypes. The highest PIC values were found in primers such as BMAC0154, BMAG0508A, BMAG0323, EBMAC0775, BMAG0131, BMAG0674, GBM1221, GBM1461, and SCSSR04163 (Shirvani *et al.* 2024).

**Figure 4.**
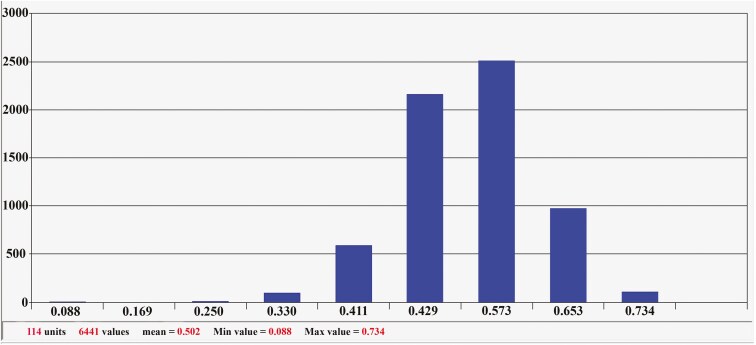
Distribution charts of the frequency of genetic distances between the examined genotypes based on SSR and EST-SSR markers.

The genetic similarity of the studied genotypes was calculated using the Jaccard coefficient, and a dissimilarity matrix for these genotypes was constructed. The average genetic distance among the genotypes was 0.502, indicating that the distances between the genotypes using SSR and EST-SSR markers ranged from 0.088 to 0.734. The greatest distance was observed between genotypes 1 Mahidasht (Kermanshah) and 12 Dehloran (Ilam) (0.734), while the smallest distance was between genotypes 103 Kermanshah (Kermanshah) and 104 Kouhdasht (Lorestan) (0.088). The frequency distribution chart showed that the maximum distance among the genotypes was 0.573 and the minimum distance was 0.088 (Figure 4).

**Figure 5.**
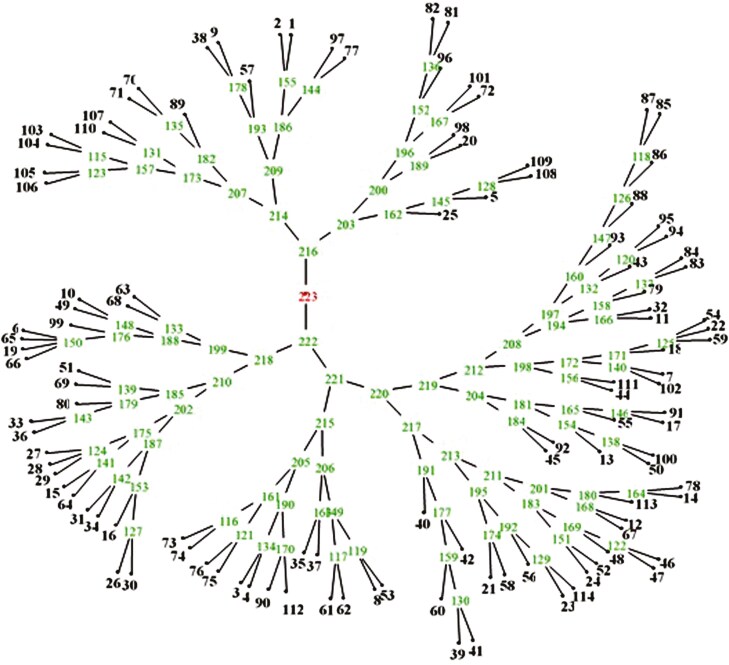
Cluster analysis resulting from grouping wild barley genotypes using SSR and EST-SSR markers with the UPGMA method. The green numbers on the figure indicate the relationship and genetic distance between the studied genotypes. The closest genotypes are placed next to each other based on the Jaccard distance coefficient, resulting in their merger and the formation of a new cluster.

The congruence of the dendrograms with the dissimilarity coefficient matrices was measured using cophenetic correlation. Cophenetic coefficients between the dissimilarity matrices of Dice, Jaccard, and Simple Matching were calculated using the UPGMA algorithm. The results indicated that for SSR and EST-SSR markers, the highest coefficient corresponded to the Jaccard dissimilarity matrix with a value of 0.82. Based on this, cluster analysis was performed for both types of markers (Figure 5). According to the EST-SSR and SSR markers, the genotypes were classified into 9 groups. Group one included 11 genotypes, group two included 6 genotypes, group three included 9 genotypes, group four included 15 genotypes, group five included 14 genotypes, group six included 20 genotypes, group seven included 8 genotypes, group eight included 8 genotypes, and group nine included 13 genotypes.

**Figure 6.**
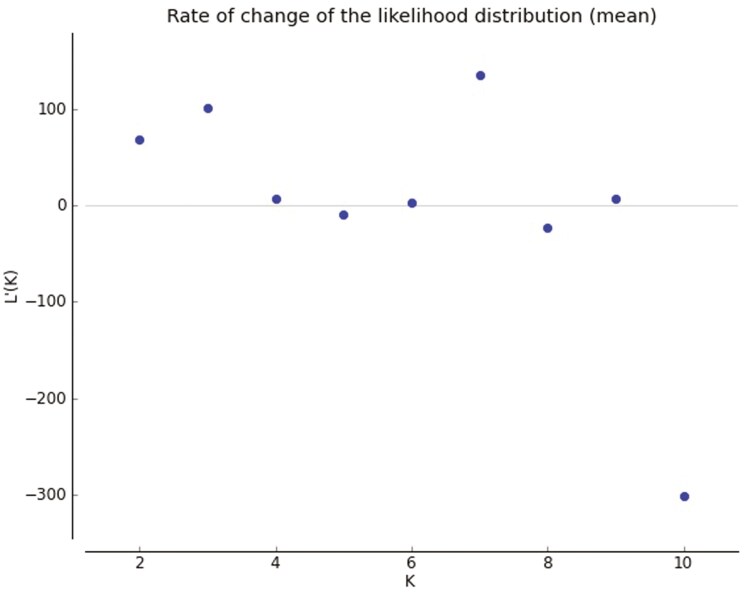
Delta *K* values calculated by Evanno’s method showing a peak at *K* = 7.

**Figure 7.**
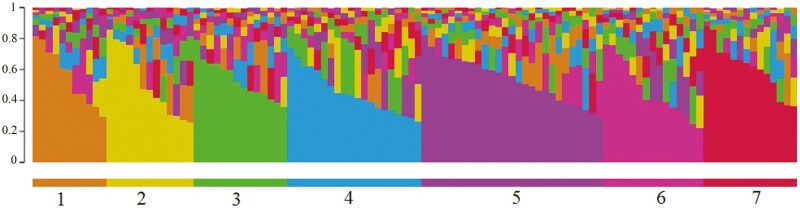
Shows Evanno results based on the Bayesian model for 114 wild barley genotypes studied based on 35 microsatellite locations (*K* = 7).

Based on the Δ*K* and LnP(D) indices, the most likely value of *K* for the genotypic grouping was found to be 7 (Figure 6). This value was considered as the optimal *K* for estimating population structure and calculating the membership matrix (*Q* matrix) for each cluster (Figure 7). The groupings obtained from the Bayesian method, based on Hardy–Weinberg equilibrium within each subpopulation, were somewhat different from those derived from the cluster analysis. This discrepancy was due to the different estimates of genetic diversity between families provided by the two systems.

Subgroup third had the highest heterozygosity (0.286), while subgroup fourth exhibited the lowest heterozygosity (0.126). Additionally, subgroup fourth showed the highest Fst value, whereas subgroup seventh had the lowest (Table 4).

**Table 4.** Mean expected heterozygosity and Fst between individuals in each of the barplot subgroups.

	First subgroup	Second subgroup	Third subgroup	Fourth subgroup	Fifth subgroup	Sixth subgroup	Seventh subgroup
He	0.223	0.2199	0.286	0.126	0.2051	0.2135	0.2534
Fst	0.4424	0.3639	0.3344	0.6779	0.4845	0.4181	0.3339

**Figure 8.**
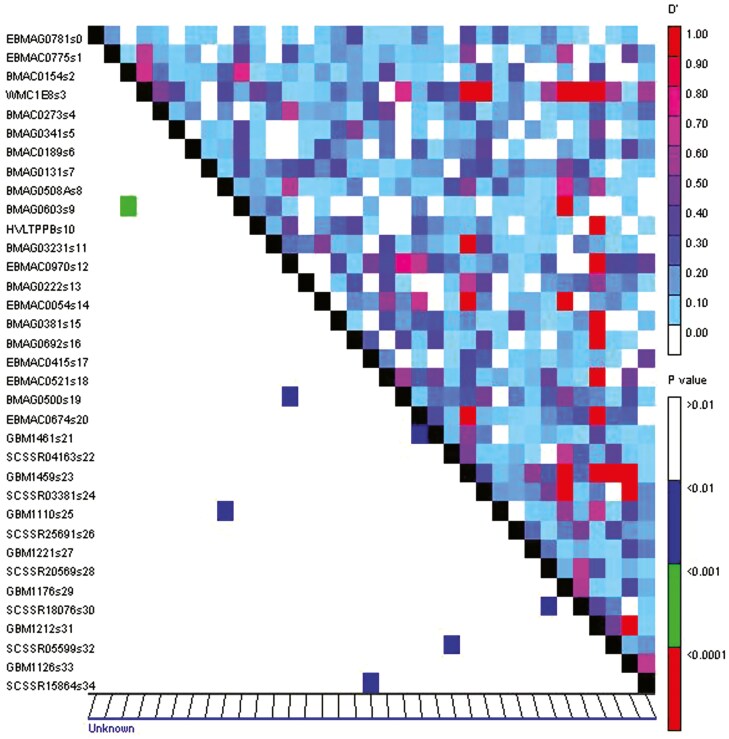
Linkage disequilibrium plot based on SSR and EST-SSR markers. The upper diagonal part shows the degree of linkage disequilibrium using the D’ statistic, while the lower diagonal part presents the *P*-value for the pair of markers. The *X*-axis represents the position of the SSR and EST-SSR markers along the chromosomes, with ‘unknown’ indicating markers for which the exact chromosomal position is not available or was not determined. The *Y*-axis represents the markers themselves. The linkage disequilibrium between each pair of markers is calculated and represented accordingly.

Linkage disequilibrium (LD) was calculated using data from 97 amplified alleles at 35 marker loci, and a Manhattan plot was generated using Tassel 3.0 software. The LD analysis between the evaluated markers is illustrated in Figure 8, showing a high level of linkage disequilibrium across the wild barley genome. This high LD is a prerequisite for analyzing the association of evaluated markers with traits in each genotype.

### Association analysis

The findings presented in Table 5 underscore the significant Marker-Trait Associations (MTAs) identified between SSR and EST-SSR markers and various physiological, seedling, and root traits. Additionally, Table 5 elucidates these associations by providing significant F-values, p-values, and R² values for each trait-marker relationship under both water-stress and normal conditions.

**Table 5.** Marker-Trait Association (MTAs) between SSR and EST-SSR markers and root, seedling, and physiological traits under water stress and normal conditions using mixed linear model (MLM).

Normal condition					Water stress				
Traits	Locus	*F*	*P*	*R* ^2^	Traits	Locus	*F*	*P*	*R* ^2^
Seedling dry weight					Seedling dry weight	BMAG0189	8.229	0.005	0.174
	HVLTPPB	7.406	0.008	0.166		HVLTPPB	7.308	0.008	0.166
	EBMAC0521	6.482	0.012	0.158		BMAG0323	6.437	0.013	0.158
	BMAG0189	3.986	0.048	0.136		SCSSR15864	4.936	0.028	0.145
	EBMAG0781	3.976	0.049	0.136		SCSSR03381	4.696	0.032	0.142
						SCSSR25691	4.527	0.036	0.141
						BMAG0341	4.207	0.043	0.138
Root dry weight					Root dry weight	BMAC0154	7.195	0.008	0.164
						BMAG0603	7.014	0.009	0.163
	EBMAC0521	6.949	0.01	0.162		SCSSR20569	5.785	0.018	0.152
	BMAG0500	5.926	0.017	0.153		BMAG0603	5.498	0.021	0.149
	GBM1126	5.904	0.017	0.152		GBM1126	4.808	0.03	0.143
						SCSSR15864	4.173	0.043	0.137
						BMAG0222	4.032	0.047	0.136
Seedling fresh weight	GBM1126*	4.086	0.046	0.139	Seedling fresh weight	GBM1126*	7.888	0.006	0.171
						EBMAC0674	4.678	0.033	0.142
Root fresh weight	EBMAC0775	7.118	0.009	0.163	Root fresh weight	GBM1176	11.367	0.001	0.2
	WMC1E8	5.275	0.024	0.147		GBM1221	6.779	0.011	0.16
	WMC1E8	5.275	0.024	0.147		SCSSR05599	4.678	0.033	0.141
	BMAG0323	4.388	0.039	0.139					
Root diameter					Root diameter	SCSSR04163	9.046	0.003	0.175
	EBMAC0521	6.369	0.013	0.153		SCSSR25691*	7.724	0.006	0.164
	BMAC0273	5.056	0.027	0.142		SCSSR04163	6.122	0.015	0.151
	BMAC0273	4.26	0.041	0.136		GBM1221	5.959	0.016	0.15
	SCSSR25691*	4.078	0.046	0.134		GBM1126	5.68	0.019	0.147
						SCSSR25691	5.097	0.026	0.142
						GBM1126	5.006	0.027	0.142
Specific root length	EBMAC0521	5.163	0.025	0.147	Specific root length	GBM1126	5.902	0.017	0.152
	SCSSR25691	4.627	0.034	0.142		SCSSR04163	5.263	0.024	0.147
	BMAG0692	4.034	0.047	0.136		SCSSR03381	4.542	0.035	0.14
						GBM1221	4.05	0.047	0.136
Seedling length					Seedling length	GBM1110	7.031	0.009	0.161
	GBM1126	4.338	0.04	0.135		SCSSR25691	6.744	0.011	0.159
						GBM1126	4.214	0.042	0.137
Root length	EBMAC0775	7.118	0.009	0.163	Root length	GBM1176	11.364	0.001	0.2
	WMC1E8	5.275	0.024	0.147		GBM1221	6.776	0.011	0.16
	WMC1E8	5.275	0.024	0.147		SCSSR05599	4.678	0.033	0.141
	BMAG0323	4.388	0.039	0.139					
Root fineness					Root fineness	EBMATC0054	6.929	0.01	0.159
						SCSSR25691	5.67	0.019	0.148
	BMAG0131	8.529	0.004	0.172		EBMATC0054	4.713	0.032	0.14
	SCSSR25691	3.991	0.048	0.134		GBM1459	4.262	0.041	0.136
						SCSSR15864	4.173	0.043	0.135
						SCSSR15864	3.968	0.049	0.134
Root area	BMAG0131	5.495	0.021	0.148	Root area	BMAG0603	10.332	0.002	0.189
	HVLTPPB*	5.035	0.027	0.144		BMAG0603	6.267	0.014	0.154
	EBMAC0674	4.478	0.037	0.139		BMAG0692	6.246	0.014	0.154
	SCSSR15864	4.165	0.044	0.136		HVLTPPB*	6.025	0.016	0.152
Root specific mass	GBM1176	6.965	0.01	0.165	Root specific mass	BMAG0500	5.941	0.016	0.151
	BMAC0273	4.905	0.029	0.145		SCSSR04163	5.198	0.025	0.144
	SCSSR05599	4.405	0.038	0.141		GBM1110	4.954	0.028	0.142
Root volume	BMAG0603	6.228	0.014	0.151	Root volume	BMAG0603*	9.124	0.003	0.181
	BMAG0603*	5.774	0.018	0.148		BMAC0154	8.614	0.004	0.176
	EBMAC0674	4.602	0.034	0.138		BMAG0603	7.941	0.006	0.17
	BMAG0131	4.359	0.039	0.136		SCSSR18076	4.231	0.042	0.137
						SCSSR20569	4.187	0.043	0.137
Root length density					Root surface area density	EBMAC0970	7.29	0.008	0.166
						EBMAC0970	7.29	0.008	0.166
						SCSSR18076	6.944	0.01	0.162
	EBMAC0674	5.445	0.021	0.149		BMAG0692	4.059	0.046	0.136
						GBM1176	7.528	0.007	0.168
						BMAG0323	6.363	0.013	0.158
						BMAG0323	4.133	0.044	0.137
Root mass density	BMAG0603*	6.23	0.014	0.151	Root mass density	BMAG0603*	9.115	0.003	0.181
	BMAG0603*	5.775	0.018	0.148		BMAC0154	8.607	0.004	0.176
	EBMAC0674	4.601	0.034	0.138		BMAG0603*	7.933	0.006	0.17
	BMAG0131	4.356	0.039	0.136		SCSSR18076	4.228	0.042	0.137
				0.1		SCSSR20569	4.189	0.043	0.137
Root tissue density					Root tissue density	SCSSR04163	8.069	0.005	0.164
						SCSSR25691*	6.18	0.014	0.149
	EBMAC0521	7.672	0.007	0.17		SCSSR04163	5.733	0.018	0.146
	SCSSR25691*	6.216	0.014	0.156		GBM1221	5.587	0.02	0.145
	SCSSR25691	4.864	0.03	0.144		GBM1126	5.533	0.02	0.144
						SCSSR25691	5.147	0.025	0.141
						BMAG0508A	4.958	0.028	0.14
Total chlorophyll	BMAG0500	9.387	0.003	0.176	Total chlorophyll				
	EBMAC0521	9.013	0.003	0.173					
	BMAG0603	6.616	0.011	0.154		GBM1176	46.709	0	0.504
	BMAG0603	6.441	0.013	0.152		HVLTPPB	5.231	0.024	0.145
	EBMAC0674	6.205	0.014	0.151		BMAC0154	4.315	0.04	0.137
	EBMAC0521	4.31	0.04	0.135					
	BMAG0341	4.173	0.043	0.134					
	GBM1126	3.937	0.05	0.132					
Chlorophyll b	GBM1126*	4.086	0.046	0.139	Chlorophyll b	GBM1126*	7.877	0.006	0.171
						EBMAC0674	4.68	0.033	0.142
Chlorophyll a	EBMAC0521	7.147	0.009	0.16	Chlorophyll a	BMAG0222	5.71	0.019	0.15
	BMAG0131	6.616	0.011	0.156		SCSSR25691	4.879	0.029	0.143
	BMAC0154	5.029	0.027	0.142		BMAG0222	4.207	0.043	0.137
Carotenoid	SCSSR20569	5.17	0.025	0.141	Carotenoid	BMAC0154	5.931	0.017	0.154

*: Common MTAs for the examined traits in both water stress and normal conditions.

The relationship between root traits and amplified alleles under water stress conditions (50-55% FC) and normal conditions has revealed significant associations between various markers and important plant characteristics, such as seedling and root dry weight, seedling fresh weight, root diameter, and physiological traits like chlorophyll content and carotenoid levels. Under water stress, markers like BMAG0189, HVLTPPB, and SCSSR15864 showed strong associations with seedling dry weight, while markers such as BMAC0154, BMAG0603, and SCSSR20569 were linked to root dry weight. Seedling fresh weight was associated with GBM1126 and EBMAC0674, and root fresh weight showed connections to markers like GBM1176 and SCSSR05599. Markers SCSSR04163 and SCSSR25691 demonstrated associations with root diameter under stress, while root vigor and specific root traits also showed notable associations with several markers, including EBMATC0054, SCSSR15864, and GBM1221.

Under normal conditions, associations also emerged between markers and root traits. For instance, markers HVLTPPB, EBMAC0521, and BMAG0189 were linked to seedling dry weight, and EBMAC0521 and BMAG0500 were connected to root dry weight. Specific root length and seedling length were influenced by markers like EBMAC0521 and GBM1126. Total chlorophyll content, which plays a crucial role in photosynthesis, showed associations with markers such as BMAG0500 and EBMAC0521. Chlorophyll a and b levels were similarly influenced by several markers, indicating their genetic control under different environmental conditions.

Root surface area density was associated with marker EBMAC0674 (allele 1). Root volume density was associated with markers BMAG0603 (allele 1), BMAG0603 (allele 2), EBMAC0674 (allele 1), and BMAG0131 (allele 3). Root tissue density was associated with markers EBMAC0521 (allele 1), SCSSR25691 (allele 2), and SCSSR25691 (allele 3). Total chlorophyll content was associated with markers BMAG0500 (allele 3), EBMAC0521 (allele 2), BMAG0603 (allele 1), BMAG0603 (allele 2), EBMAC0674 (allele 2), EBMAC0521 (allele 1), BMAG0341 (allele 2), and GBM1126 (allele 3). Chlorophyll b was associated with marker GBM1126 (allele 1). Chlorophyll a was associated with markers EBMAC0521 (allele 1), BMAG0131 (allele 3), and BMAC0154 (allele 2). Carotenoid content was associated with marker SCSSR20569 (allele 2) (Table 5).

**Figure 9.**
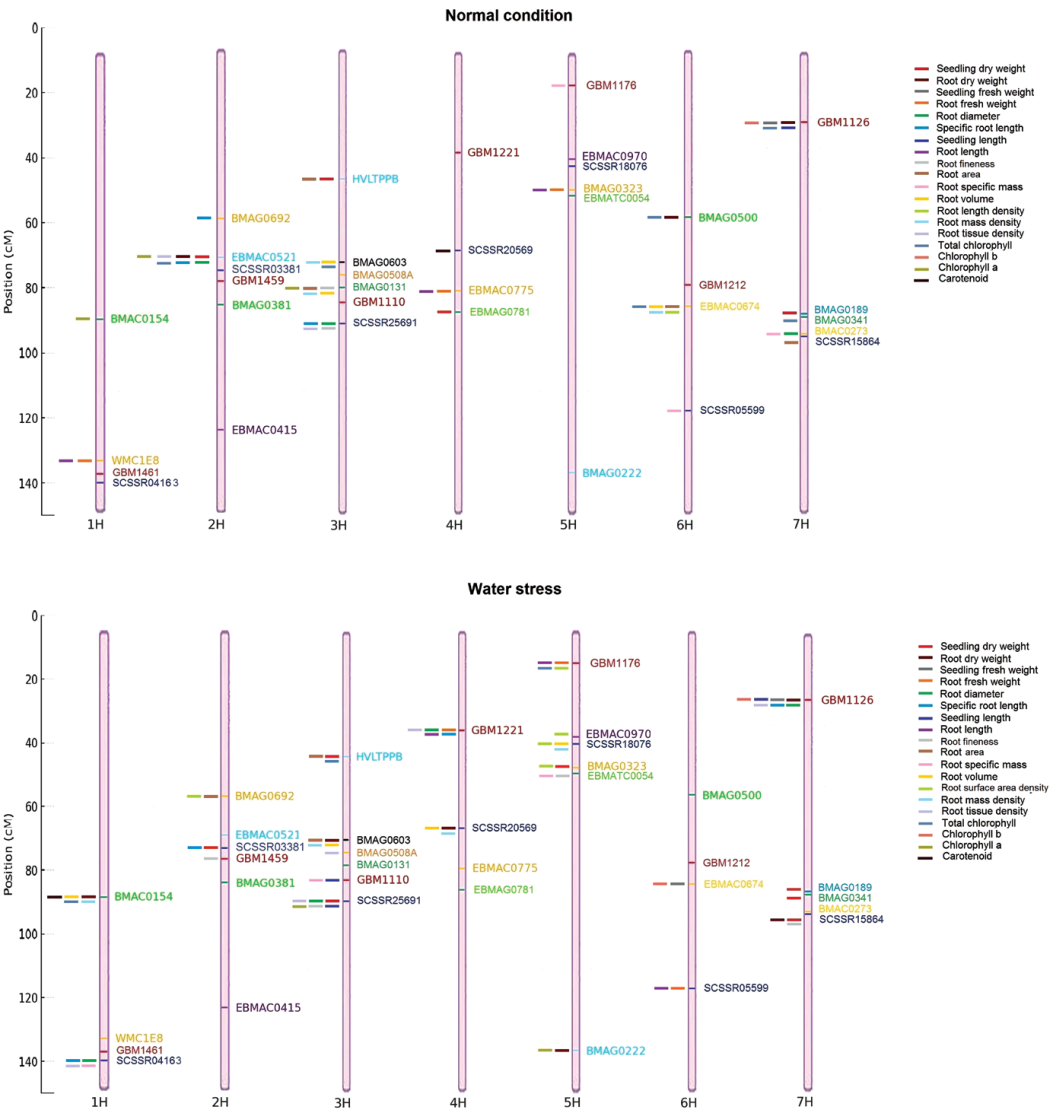
The position of markers used on each chromosome and their relationship with the investigated traits in both normal and water stress conditions. The figure was produced using the ggplot2 software package in the R programming, with marker position data sourced from the GrainGenes database (https://wheat.pw.usda.gov/GG3/).

The positions of the markers used on each chromosome are shown in Figure 9. The traits related to the markers are also displayed on this figure next to the marker. The chromosomal mapping results indicate that under both normal and water-stress conditions, various markers were associated with traits related to seedling growth and root characteristics, such as seedling dry and fresh weight, root length, root tissue density, and chlorophyll content. Notably, under water-stress conditions, markers located on linkage groups 1H, 2H, and 5H were predominantly associated with root length and root density traits, suggesting that these chromosomal regions play a critical role in the plant’s response to water stress. Additionally, some markers, such as those linked to root density and chlorophyll content, were identified under both normal and water-stress conditions, indicating the genetic stability of these regions.

## Discussion

The results of this study highlight significant diversity in root traits among different genotypes under water stress and normal conditions. Categorizing genotypes based on root tissue density and root length provided a deeper understanding of the impact of water stress on various root characteristics. Under water stress conditions, the mean values for all root traits, except for fresh root weight, root surface area, and specific root length, decreased. This indicates a negative effect of water stress on many root traits, especially those related to storage and water absorption. This reduction can be attributed to the plant’s diminished ability to absorb and retain water during dry periods, which, in turn, affects root growth and development. These findings align with those of Halušková *et al.* who emphasized the impact of environmental factors such as drought and salinity on root health and root degradation rates in barley plants. (Halušková *et al.* 2010). Additionally, Ramireddy *et al.* focused on the genetic aspects, identifying key genes that regulate root development and stress responses, which could be targeted for enhancing drought tolerance (Ramireddy *et al.* 2018).

Genetic diversity analysis using SSR and EST-SSR markers revealed a high level of variability among the genotypes, identified through the number and types of alleles and the high percentage of polymorphism. This genetic diversity serves as a valuable resource for identifying markers linked to desirable traits under normal and water stress conditions. Cluster analysis, conducted using the WARD method, along with population structure analysis using the Structure software, revealed a clear division of genotypes into distinct groups. These analyses demonstrated significant genetic structural differences among these groups, which can enhance our understanding of the mechanisms involved in stress response and improve breeding strategies. The linkage disequilibrium analysis indicated a high degree of linkage across the genome, providing a foundation for marker-trait association studies. Similar results were reported by Hübner *et al.* who identified seven wild barley groups based on different sampling locations in Israel using 42 SSR markers and highlighted the influence of abiotic factors such as altitude, temperature, and precipitation on genetic differentiation between populations (Hübner *et al.* 2009).

The marker-trait association analysis was a vital tool in identifying molecular markers linked to specific phenotypic traits. This study showed that several SSR and EST-SSR markers were consistently associated with various root traits and physiological traits under both water stress and normal conditions. These findings underscore the importance of identifying key markers that could improve traits related to drought tolerance. Marker-trait association analysis for root traits in wild barley indicated substantial genetic variation and the potential to enhance drought tolerance. A study using a mapping population (HEB-25) identified 52 QTLs associated with root architecture traits under controlled and osmotic stress conditions (Khodaeiaminjan *et al.* 2023). Additionally, another research effort within the HEB-25 population found that wild barley alleles could enhance biomass under water stress conditions, with specific QTL identified on chromosome 4H (Pham *et al.* 2019). Furthermore, a genome-wide association study on a diverse spring barley collection uncovered 34 root-specific loci and several QTL hotspots, emphasizing the polygenic nature of root architecture (Abdel-Ghani *et al.* 2019). Overall, these findings underscore the potential of wild barley as a genetic resource for targeted breeding programs aimed at improving root traits and drought tolerance in cultivated barley (Sharma *et al.* 2018; Pham *et al.* 2024).

Under water stress conditions, several markers were associated with different root traits such as seedling dry weight, root dry weight, fresh root weight, root diameter, and root volume. These associations suggest that certain markers can be used to identify genotypes with desirable traits, such as higher dry weight, under water stress. The results for all traits under both water stress and normal conditions showed that alleles 1 and 2 of the BMAG0603 marker were most frequently associated with 15 different traits, totaling 30 traits. In contrast, markers BMAG0189 (alleles 1 and 3), BMAG0131 (alleles 2 and 3), BMAG0341 (alleles 1 and 2), BMAG0500 (allele 1), BMAG0508A (allele 1), EBMATC0054 (allele 2), EBMAC0674 (allele 2), EBMAC0970 (alleles 1 and 2), EBMAG0781 (allele 1), GBM1221 (allele 2), GBM1459 (allele 1), HVLTPPB (alleles 1 and 3), and SCSSR20569 (allele 2) were less frequently associated with traits, each being linked to only one trait. A GWAS study identified 17 QTLs for root and shoot traits, with significant loci on chromosome 1H affecting root dry weight and stem number (Reinert *et al.* 2016). Another study validated wild alleles in the cultivated barley background and identified significant QTLs for root dry weight, root volume, and stem number on chromosome 5H (Ahmad Naz *et al.* 2012).

Under normal conditions, similar marker-trait associations were observed, although with differences in the strength and nature of these associations. These findings indicate that certain markers can be used to identify genotypes with desirable traits under normal conditions. The results showed that markers GBM1126 (allele 1), SCSSR25691 (allele 2), HVLTPPB (allele 4), and BMAG0603 (alleles 1 and 2) were consistently associated with traits such as seedling fresh weight, root diameter, root surface area, root volume, root tissue density, root tissue density, and chlorophyll b under both water stress and normal conditions. A study on spring barley identified 55 QTLs associated with root traits such as root system depth, root spreading angle, and seminal root length. Major QTLs were found on chromosomes 2H and 3H, explaining significant phenotypic variations (Jia *et al.* 2019). Research on two-row spring barley landraces under different water conditions identified 276 significant marker-trait associations for root and shoot traits. This study highlighted the genetic diversity present within the landraces and identified several QTLs associated with root system architecture under stress condition (Khodaeiaminjan *et al.* 2023).

Comparing the results under water stress and normal conditions reveals that some markers, such as BMAG0603 and GBM1126, were consistently associated with various root and physiological traits. This consistency in marker-trait associations suggests that certain root traits and physiological traits are genetically stable, which can aid breeding programs aiming to improve drought tolerance. Specifically, markers BMAG0603 (alleles 1 and 2) and SCSSR25691 (allele 2) were linked to a large number of traits, making them strong candidates for improving desirable traits. These findings provide valuable insights into the role of specific markers in enhancing drought tolerance and offer promising targets for genetic improvement in barley breeding. A genome-wide association study (GWAS) identified several associations between root- and yield-related traits and single-nucleotide polymorphism (SNP) markers, as well as linkage disequilibrium (LD) blocks, for 11 phenotypic traits in spring barley accessions (Ogrodowicz *et al.* 2023).

The results from the marker mapping under normal and water-stress conditions reveal that the plant’s response to water stress is influenced by various genes and chromosomal regions. The identification of multiple markers associated with root traits under water-stress conditions highlights the importance of the root system in coping with water scarcity. The concentration of markers related to root traits on linkage groups 1H and 2H suggests the presence of crucial genes in these regions that regulate the plant’s drought response. Furthermore, the identification of markers common to both normal and water-stress conditions indicates that certain growth and root density traits are under stable genetic control. These traits could be targeted in breeding programs aimed at improving drought tolerance in plants. The absence of markers in certain regions can be attributed to the limited coverage of the markers used in this study, which may not fully capture the genetic diversity present across the entire genome. Additionally, it is possible that these marker-free regions are less polymorphic or that the specific markers chosen were not optimal for detecting variability in these segments. It is important to note that large chromosomal regions, including those without markers, may still harbor valuable candidate genes for drought tolerance and other traits. However, identifying these genes may require the application of additional markers or advanced genomic techniques in future studies.

## Conclusion

This study, conducted on a germplasm collection of 114 wild barley genotypes from the natural distributional range in the western provinces of Iran, underscores the significant relationships between molecular markers and root traits under both water stress and normal conditions. The analyses using SSR and EST-SSR markers revealed a high level of genetic diversity among the genotypes, with significant associations between these markers and various root characteristics. This classification provided valuable insights into the relationships between markers and root traits, enabling a more detailed examination of the effects of water stress on root development.

The association analysis highlighted that certain markers, including BMAG0603 and GBM1126, were consistently linked to desirable root traits such as seedling dry weight, root dry weight, root length, root diameter, and root volume. These markers can serve as key indicators for improving root traits and developing drought-resistant genotypes. The statistical significance of these associations was confirmed through rigorous analysis, further strengthening their potential utility in breeding programs.

Under water stress conditions, the relationships between markers and root traits varied, with some markers showing stronger associations with specific traits such as dry weight and root volume. This suggests that these markers may play a critical role in identifying traits related to drought tolerance and optimizing water management strategies in stress conditions. Future studies should focus on evaluating the consistency of these markers across different environmental conditions to better understand their potential for widespread application in breeding programs.

It is recommended that the identified key markers, particularly BMAG0603 and GBM1126, be utilized as essential tools in breeding programs aimed at developing drought-resistant genotypes and enhancing root traits. Further research could explore how these markers perform in combination with other agronomic traits to improve the overall drought tolerance of barley.

These findings and recommendations offer significant implications for advancing genetic improvement programs and developing effective water management strategies. They provide a foundation for breeding drought-resistant barley varieties, contributing to the adaptation of barley cultivation in dry and semi-dry environments. Additionally, the study highlights the need for further exploration of unmarked genomic regions that may harbor valuable candidate genes for drought tolerance and other important traits, which could be investigated using advanced genomic tools in future studies.

## Supplementary Material

plaf022_suppl_Supplementary_Tables_S1-S2_Figures_S1

plaf022_suppl_Supplementary_File

## Data Availability

The data underlying this article are available in Zenodo at https://doi.org/10.5281/zenodo.15034069.
