# Supplementary material for: Marker-trait association analysis for root and shoot traits at the seedling stage of wild barley (Hordeum vulgare subsp. spontaneum) under water stress and normal conditions
Source: AoB Plants. 2025 Jul 16;17(4):plaf022. doi: 10.1093/aobpla/plaf022 (PMC12264725; doi:10.1093/aobpla/plaf022)
Supplement: plaf022_suppl_Supplementary_File [file plaf022_suppl_supplementary_file.docx]

**Dear Editor,**

I hope this message finds you well.

In response to the reviewer’s comments, it appears that they believe the traits measured in our study do not directly reflect the primary drought tolerance mechanisms of the plant, but instead are more related to structural ratios and plant size. Based on this evaluation, we would like to address the points raised and clarify the relationship between the selected traits and drought stress, as follows:

1. **Justification for Trait Selection and Their Relevance to Drought Stress:** In the Introduction and Discussion sections, we have provided a scientific explanation for the selection of these traits and discussed their relationship with drought tolerance, citing relevant literature. For instance, traits such as root diameter and root density are related to the plant's ability to absorb water and nutrients from the soil under stress conditions. Specifically, root tissue density, root mass density, and specific root surface area may indirectly indicate the plant's ability to penetrate dry soils and its resistance to water scarcity.
2. **Comparison with Recent Studies:** In the Discussion section, we have compared our results with recent studies that have linked similar traits to drought tolerance. These references help to strengthen the significance of our findings and place them within the broader context of drought research.
3. **Additional Analyses:** Given the available data, we have applied more advanced statistical methods to analyze the correlation between these traits and drought stress indices (such as drought sensitivity indices and plant performance under water-limited conditions).
4. **Expansion of the Discussion and Results Section:** The analyses presented in the Discussion have been elaborated to further highlight the importance of these traits in drought tolerance. For example, if root mass density is higher in drought-tolerant plants, it can be considered an indicator of the plant’s strategy to optimize water use.
5. **Marker-Trait Association Analysis:** Additionally, a marker-trait association analysis was conducted in our study, which is one of the novel aspects of this work. The analysis revealed significant associations between molecular markers and root traits under both drought and normal conditions. These findings are crucial for identifying genetic markers linked to drought tolerance, offering potential tools for future breeding programs aimed at improving drought resistance in barley genotypes.

We hope that these revisions and explanations will address the reviewer’s concerns and enhance the scientific contribution of the manuscript. Thank you for your time and consideration.

Sincerely,
